# A longitudinal study on the association between quick returns and occupational accidents

**DOI:** 10.5271/sjweh.3906

**Published:** 2020-10-30

**Authors:** Øystein Vedaa, Anette Harris, Siri Waage, Bjørn Bjorvatn, Eirunn Thun, Hogne Vikanes Buchvold, Ingebjørg Louise Rockwell Djupedal, Ståle Pallesen

**Affiliations:** 1 Department of Health Promotion, Norwegian Institute of Public Health, Bergen, Norway; 2 Department of Mental Health, Norwegian University of Science and Technology, Trondheim, Norway; 3 Voss District Psychiatric Hospital, NKS Bjørkeli AS, Voss, Norway; 4 Department of Psychosocial Science, University of Bergen, Bergen, Norway; 5 Department of Global Public Health and Primary Care, University of Bergen, Bergen, Norway; 6 Norwegian Competence Center for Sleep Disorders, Haukeland University Hospital, Bergen, Norway

**Keywords:** fatigue, nurse, occupational injury, sleep, sleepiness, shift work, shift worker

## Abstract

**Objective:**

This study aimed to investigate how change in the number of quick returns [(QR) <11 hours between consecutive shifts] longitudinally is associated with risk of occupational accidents among nurses.

**Methods:**

Two-year follow-up data from 1692 nurses participating in the Survey of Shiftwork, Sleep and Health among Norwegian nurses (SUSSH) (mean age 40.2, standard deviation 8.3 years, 91% female) were used. Negative binomial regression analyses were conducted to investigate the association between changes in the number of QR after two years and occupational accidents, controlling for demographics, work factors, and occupational accidents at baseline.

**Results:**

An increase from having no or a moderate number of QR (1–34 per year) from baseline to the two-year follow-up assessment was associated with an increased risk of occupational accidents, compared to experiencing no change in the number of QR. Those with a moderate number of QR at baseline who experienced an increase after two years had an increased risk of causing harm to patients/others [incident rate ratio (IRR) 8.49, 95% confidence interval (CI) 2.79–25.87] and equipment at work (IRR 2.89, 95% CI 1.13–7.42). Those who had many QR (>34 per year) at baseline but experienced a reduction after two years had a reduced risk of causing harm to themselves (IRR 0.35, 95% CI 0.16–0.73) and patients/others (IRR 0.27, 95% CI 0.12–0.59).

**Conclusion:**

A fairly consistent pattern was demonstrated in which changes in the number of QR over the two-year follow-up period was associated with a corresponding change in the risk of occupational accidents.

Twenty-three percent of employees in European countries reported having at least one quick return [(QR) <11 hours of rest between two consecutive work shifts] in the last month (1). QR are associated with short sleep duration, increased daytime sleepiness and fatigue (2, 3). This is assumed to be the central mechanism for the empirically supported link between QR and risk of injury and occupational accidents (4–7). In a study where objective data on exposure to QR (payroll data) was linked to national records of injuries (N>60 000), it was demonstrated that QR were associated with a 39% higher risk of injury compared to having 15–17 hours off between shifts (4). Based on the same register data, it was shown that QR are primarily associated with occupational injuries, rather than injuries that occur during commutes or leisure time (7). In a recent study based on a cross-sectional survey, QR were associated with a higher risk of nurses reporting causing harm to themselves, patients/others, and equipment at work (6). However, the temporal relationship between QR and accidents and injuries has yet to be investigated, specifically whether a reduction or an increase in the number of QR over time are associated with a corresponding change in the risk of such incidences.

Accordingly, the aim of the present study was to investigate how a reduction or an increase in the number of QR over time are associated with the risk of nurses reporting occupational accidents. We hypothesized that a reduction or an increase in the number of QR over time would be associated with a corresponding decrease and increase in the risk of occupational accidents.

## Methods

### Study design

We used data from the Survey of Shiftwork, Sleep and Health (SUSSH) among Norwegian nurses. The SUSSH cohort was initiated in the winter season of 2008/2009, and members of the Norwegian Nurses Organisation (N=6000) were asked to participate. They were randomly selected from five different strata based on number of years since they qualified as a nurse. We invited 1200 nurses from each of the following five strata: 0–1.0, 1.1–3.0, 3.1–6.0, 6.1–9.0, and 9.1–12.0 years since completing the degree, respectively; and 600 invitations were returned due to wrong addresses. Finally, 2059 nurses responded (response rate=38% [2059/5400]). Later in 2009, 2741 newly graduated nurses were invited to join the cohort, of which 905 agreed (response rate=33%). These two groups formed the baseline cohort of the SUSSH, for which there since have been annual data collections. The present study used data from the 2016 (N=1841; response rate=66%) and the 2018 (N=1698; response rate=66%) waves.

The survey questionnaire was sent to each nurse’s home address by letter (with two reminders to non-responders), together with a prepaid return envelope. Participation was voluntary, and the Regional Committee for Medical Research Ethics in Western Norway (No. 088.88) and the Norwegian Data Inspectorate (08/01235/IUR) approved the study protocol.

### Questionnaires

Information about the participants’ sex and age was assessed in 2008/09 (participants’ age in 2018 was calculated based on that information). Relationship status and child care responsibility (in the household; yes/no) were assessed in 2018. Participants were asked about their percentage of full time equivalent (FTE) in 2018, which had the four response categories: <50%, 50–75%, 76-90% and >90%.

*Shift work*. Exposure to QR was assessed both in 2016 and 2018 with the open-ended question: “Over the past 12 months, how many times have you had <11 hours free between two consecutive shifts?” Exposure to night shift was assessed in 2018 with the question: “How many night shifts have you worked in the last 12 months?”

*Occupational accidents*. Three items assessing the number of self-reported occupational accidents were constructed for the purpose of the SUSSH cohort (6, 8) and included in both the 2016 and 2018 surveys. The questions were open-ended and phrased as follows: “How many times during the last year have you: 1) Experienced occupational accidents that you felt were your fault, causing harm to yourself? 2) Experienced occupational accidents you felt were your fault, causing harm to patients/others? 3) Experienced occupational accidents you felt were your fault, causing harm to equipment?”

### Statistical analysis

SPSS Statistics (version 25 for Macintosh, IBM, Armonk, NY, USA) was used to run GLM negative binomial regression in order to investigate the relationship between changes in number of QR from 2016 to 2018 and occupational accidents in 2018. The number of participants in the different analyses varied due to missing data. Occupational accidents are generally rare events with a mean close to 0 (skewed distribution), in which negative binomial regression had the best model fit. We separated between those who had no QR, a moderate number (1–34), and a high number (>34) in 2016, which created three groups approximately equal in terms of number of participants. Further, for each of these three groups, as a measure of change in number of QR the last year in 2016 to the number of QR the last year in 2018, we separated between those who had no change in the number of QR (“no change”; allowing for a margin of +/-4 QR for the “no change” category), those who had a reduction in the number of QR (“reduction”), and those who had an increase in the number of QR (“increase”). Thus, change in QR over the two-year assessment period was included as a categorical variable in the analyses. Crude models were tested, as well as models adjusting for sex, age, relationship status, having children in the household, percentage of FTE, number of night shifts the last year in 2018, and occupational accidents the past year at baseline. The rationale for including this list of confounders was that they define some of the basic elements of an individual’s life and work situation, all of which have been found to account for part of the variance of occupational accidents in previous research (eg, 6, 8).

Some participants (N=9) reported having >150 QR during the last year. These figures were deemed erroneous and replaced by 150, as we considered this the maximum number of QR one person can possibly have in one year. Results are presented as log counts and exponential parameter estimates [Exp (B)] with 95% confidence intervals (CI). Exp(B) is referred to as incidence rate ratios (IRR) in this paper. An alpha level of 0.05 was set to indicate statistical significance. Missing values were treated as invalid in the analyses.

## Results

Descriptive statistics of demographics, work factors and occupational accidents are reported in table 1. Descriptive statistics of occupational accidents across subgroups in this study are presented in the supplementary material (www.sjweh.fi/show_abstract.php?abstract_id=3906) table S1.

**Table 1:** Descriptive statistics of demographics and work factors. [FTE=full time equivalent; QR=quick return; SD=standard deviation].

Variables	Total sample (N=1692)		Classification based on number of QR in the last 12 months at baseline					

			No QR (N=537)		Moderate (1–34) QR (N=553)		High (>34) QR (N=574)	
			
	N (%)	Mean (SD)	N (%)	Mean (SD)	N (%)	Mean (SD)	N (%)	Mean (SD)
Sex								
Female	1538 (90.9)		405 (90.8)		416 (90.8)		427 (90.7)	
Male	154 (9.1)		41 (9.2)		42 (9.2)		44 (9.3)	
Age in 2018		40.2 (8.3)		42.8 (8.2)		41.1 (7.6)		42.5 (9.1)
Relationship status in 2018								
Living with partner	1358 (79.8)		361 (81.1)		374 (82.0)		368 (78.1)	
Living without partner	332 (20.2)		84 (18.9)		82 (18.0)		103 (21.9)	
Children in household in 2018								
No children	475 (28.5)		112 (26.0)		128 (28.1)		171 (36.5)	
Children in household	1194 (69.7)		318 (74.0)		327 (71.9)		298 (63.5)	
Percentage of FTE in 2018								
>90%	893 (62.5)		212 (60.4)		251 (61.8)		292 (66.8)	
76–90%	275 (19.2)		57 (16.2)		79 (19.5)		87 (19.9)	
50–75%	224 (15.7)		70 (19.9)		67 (12.1)		50 (11.4)	
<50%	37 (2.6)		12 (3.4)		9 (2.2)		8 (1.8)	
Shift work								
Night shifts in 2018		17.2 (33.3)		18.7 (44.2)		18.3 (32.1)		14.4 (21.1)
QR in 2016		27.6 (32.6)		0.0 (0.0)		14.5 (9.5)		66.2 (25.2)
QR in 2018		24.5 (31.2)		6.1 (18.5)		18.1 (22.2)		48.6 (32.6)
Change in QR								
Reduction from 2016 to 2018		-27.5 (23.1)		-		-13.7 (5.5)		-35.9 (25.3)
Increase from 2016 to 2018		27.8 (24.2)		34.8 (32.2)		26.4 (21.5)		25.1 (20.4)
Occupational accidents the past year in 2016								
Caused harm to oneself		0.1 (0.8)		0.1 (0.9)		0.1 (0.4)		0.2 (1.1)
Caused harm to patients/others		0.1 (0.7)		0.1 (0.7)		0.1 (0.5)		0.2 (0.9)
Caused harm to equipment		0.2 (1.0)		0.1 (0.7)		0.2 (0.6)		0.3 (1.6)
Occupational accidents the past year in 2018								
Caused harm to oneself		0.1 (0.5)		0.1 (0.3)		0.0 (0.29)		0.2 (0.6)
Caused harm to patients/others		0.1 (0.6)		0.1 (0.6)		0.1 (0.7)		0.1 (0.6)
Caused harm to equipment		0.1 (0.8)		0.1 (0.9)		0.1 (0.5)		0.2 (0.98)

The results from the negative binomial regression analyses in terms of incidence rate ratios (IRR) are reported in table 2 (log counts are reported in the supplementary material table S2). Those who had no QR and those who had a moderate number 1–34) at baseline – and experienced an increase in QR over the two-year assessment period – demonstrated an increased risk of causing harm to patients/others and equipment, compared to those in the no-change group. Those who had a high number of QR (>34) at baseline and experienced a reduction in QR over the assessment period, demonstrated a reduced risk of accidents compared to those in the no-change group.

**Table 2:** Results from the negative binomial regression analyses on the association between changes in number of quick returns (QRs) over time and occupational accidents. The no QR group includes those who had no QR at baseline (in 2016). The moderate group includes the first half of those who had QR at baseline. The high group includes the second half of those who had QR at baseline. Statistical significance (P<0.05) is indicated with estimates highlighted in bold.

	No QR at baseline					Moderate number of QR at baseline					High number of QR at baseline				
		
	N	IRR_crude_	95% CI	IRR_adj_^a^	95% CI	N	IRR_crude_	95% CI	IRR_adj_^a^	95% CI	N	IRR_crude_	95% CI	IRR_adj_^a^	95% CI
Caused harm to oneself the last year (incidences)															
No change in QR (reference) ^b^	345					120					64				
Reduction in QR	0					164	0.73	0.18–2.98	0.87	0.19–4.01	271	0.29	0.15–0.57	0.35	0.16–0.73
Increase in QR	70	1.64	0.63–4.29	1.61	0.47–5.51	148	2.45	0.77–7.79	2.58	0.71–9.34	102	1.04	0.53–2.01	1.16	0.57–2.37
Caused harm to patients/others the last year (incidences)														
No change in QR (reference) ^b^	345					120					64				
Reduction in QR	0					164	0.55	0.12–2.50	0.62	0.13–2.92	271	0.30	0.15–0.60	0.27	0.12–0.59
Increase in QR	70	3.45	1.66–7.16	6.03	2.12–17.10	148	8.72	3.04–24.97	8.49	2.79–25.87	102	0.75	0.36–1.55	0.68	0.31–1.51
Caused harm to equipment the last year (incidences)															
No change in QR (reference) ^b^	345					120					64				
Reduction in QR	0					164	1.59	0.59–4.29	0.55	0.17–1.83	271	0.55	0.31–1.00	1.00	0.46–2.38
Increase in QR	70	8.30	4.45–15.48	4.41	1.69–11.52	148	4.73	1.93–11.62	2.89	1.13–7.42	102	0.82	0.42–1.58	1.53	0.65–3.57

a Adjusted for sex, age, relationship status, having children in the household, percentage of full time equivalent (FTE), number of night shifts the past year as measured in 2018, and correspo nding number of accidents at baseline (in 2016).

b The "no change in QR" category includes those who had an equal number of QR (+/- 4) in 2016 and 2018.

## Discussion

This paper presents results from a two-year follow-up study investigating the risk of occupational accidents among nurses when experiencing a change in the number of QR in the work schedule. Overall, the results showed a fairly consistent pattern in which an increase in the number of QR was associated with an increased risk of accidents, while a decrease was associated with a reduced risk of accidents.

An increase in the number of QR over the two-year assessment period was associated with relative risks for work related accidents of 2.89–8.49 compared to those who did not change the number of QR. This suggests a considerable increased risk of occupational accidents as the number of QR increases. These findings are by and large in line with previous studies demonstrating an association between QR and injuries and occupational accidents (4–7). However, a unique pattern in this study was that an increase in the number of QR primarily increased the risk of accidents that involved causing harm to patients/others and equipment, rather than causing harm to oneself. It is unclear why there was no increased risk of causing harm to oneself; although we can speculate that the threshold for classifying something as harmful to oneself may be higher compared to what one considers harmful to others. Furthermore, for those who had many QR at baseline and experienced a reduction in the number of QR over the assessment period, the risk of causing harm to oneself and patients/others was significantly reduced. These findings seem reasonable since those who had a high number of QR at baseline had more than twice the reduction in QR over the two-year assessment period than those with a moderate number of QR.

The current study is the first to demonstrate that an increase or decrease in the number of QR over time is associated with a corresponding increase and decrease in the risk of occupational accidents. This takes us one step further towards probing the causal link between QR and occupational accidents. However, it will be necessary to demonstrate this association in a randomized controlled trial (eg, by reducing QR in one condition) before a causal link can be established.

The strengths of the SUSSH cohort include the relatively large sample size, the longitudinal design, and the homogenous sample, reducing the influence from occupational confounders. However, considering that there were relatively few nurses who reported having QR in this study (27.6% in 2016 and 24.5% in 2018), the subgroups that were created based on changes in the number of QR over time were relatively small, which raises the probability of false discoveries and inflated effect size estimations. Still, the validity of the results is supported by the fact that the outcomes were fairly consistent across the analyses. The small subgroups entail limitations in terms of statistical power. Another limitation concerns the uncertainty related to the representativeness of the findings, considering the relatively low response rate in the first wave of the SUSSH survey (9), and the subsequent dropout that has followed over the cohort period. It is a limitation that the study only included self-reported measures of exposure and outcome variables, which may have caused recall bias or social desirability bias. Additional limitations with the SUSSH cohort and the assessments included in the present study are discussed in more detail elsewhere (eg, 6, 10, 11).

## Supplementary material

Supplementary material
