# Supplementary material for: A longitudinal study on the association between quick returns and occupational accidents
Source: Scand J Work Environ Health. 2020 Oct 30;46(6):645–9. doi: 10.5271/sjweh.3906 (PMC7737807; doi:10.5271/sjweh.3906)
Supplement: Supplementary material [file SJWEH-46-645-S001.pdf]

A longitudinal study on the association between quick returns and occupational accidents[1]

by Øystein Vedaa, PhD,[2] Anette Harris, PhD, Siri Waage, PhD, Bjørn Bjorvatn, PhD, Eirunn Thun, PhD, Hogne Vikanes Buchvold, PhD, Ingebjørg Louise Rockwell Djupedal, MA, Ståle Pallesen, PhD

1. Supplementary tables

2. Correspondence to: Øystein Vedaa Ph.D., Department of Health Promotion, Norwegian Institute of Public Health, Zander Kaaes gate 7, 5018 Bergen, Norway. [E-mail: oystein.vedaa@fhi.no].

Table S1. Descriptive statistics of occupational accidents across subgroups

	No QRs at baseline				Moderate number of QRs at baseline				High number of QRs at baseline			
	n	Mean (SD) num. of accidents	Median num. of accidents	Range accidents	*n*	Mean (SD) num. of accidents	Median num. of accidents	Range accidents	*n*	Mean (SD) num. of accidents	Median num. of accidents	Range accidents
Caused harm to oneself the last year (num. of incidences)												
No change in QRs[1]	345	0.05 (0.28)	.00	0-2	120	0.03 (0.22)	.00	0-2	64	0.28 (0.97)	.00	0-5
Reduction in QRs	0	-	-	-	164	0.02 (0.15)	.00	0-1	271	0.08 (0.36)	.00	0-3
Increase in QRs	70	0.09 (0.33)	.00	0-2	148	0.08 (0.43)	.00	0-4	102	0.29 (0.82)	.00	0-5
Caused harm to patients/others the last year (num. of incidences)												
No change in QRs[1]	345	0.06 (0.57)	.00	0-10	120	0.03 (0.22)	.00	0-2	64	0.25 (1.29)	.00	0-10
Reduction in QRs	0	-	-	-	164	0.02 (0.17)	.00	0-2	271	0.07 (0.33)	.00	0-3
Increase in QRs	70	0.20 (0.79)	.00	0-6	148	0.29 (1.13)	.00	0-10	102	0.19 (0.64)	.00	0-5
Caused harm to equipment the last year (num. of incidences)												
No change in QRs[1]	345	0.06 (0.33)	.00	0-3	120	0.05 (0.29)	.00	0-2	64	0.31 (1.34)	.00	0-10
Reduction in QRs	0	-	-	-	164	0.08 (0.47)	.00	0-5	271	0.17 (0.82)	.00	0-10
Increase in QRs	70	0.46 (1.95)	.00	0-15	148	0.24 (0.69)	.00	0-5	102	0.25 (1.15)	.00	0-10

Note. The no QRs group includes those who had no QRs at baseline (in 2016). The moderate group includes the first half of those who had QRs at baseline (1-34 QRs the past year). The high group includes the second half of those who had QRs at baseline (>34 QRs the past year). Statistical significance ($p<.05$) is indicated with estimates highlighted in bold.

[1] The no change in QRs category includes those who had an equal number of QRs (+- 4) in 2016 and 2018.

Table S2. Results from the negative binomial regression analyses on the association between changes in number of quick returns (QRs) over time and occupational accidents

	No QRs at baseline			Moderate number of QRs at baseline			High number of QRs at baseline		
	n	Crude log count	Adjusted[1] log count	*n*	Crude log count	Adjusted[1] log count	*n*	Crude log count	Adjusted[1] log count
Caused harm to oneself the last year (num. of incidences)									
No change in QRs (ref.)[2]	345			120			64		
Reduction in QRs	0			164	-0.31	-0.14	271	**-1.25**	**-1.06**
Increase in QRs	70	0.50	0.48	148	0.89	0.94	102	0.04	0.15
Caused harm to patients/others the last year (num. of incidences)									
No change in QRs (ref.)[2]	345			120			64		
Reduction in QRs	0			164	-0.60	-0.48	271	**-1.22**	**-1.32**
Increase in QRs	70	**1.24**	**1.80**	148	**2.17**	**2.14**	102	-0.29	-0.39
Caused harm to equipment the last year (num. of incidences)									
No change in QRs (ref.)[2]	345			120			64		
Reduction in QRs	0			164	0.46	-0,59	271	-0.59	0.04
Increase in QRs	70	**2.12**	**1.48**	148	**1.55**	**1.06**	102	-0.21	0.43

Note. The no QRs group includes those who had no QRs at baseline (in 2016). The moderate group includes the first half of those who had QRs at baseline (1-34 QRs the past year). The high group includes the second half of those who had QRs at baseline (>34 QRs the past year). Statistical significance (p<.05) is indicated with estimates highlighted in bold.

[1] Adjusting for sex, age, relationship status, having children in the household, percentage of full time equivalent (FTE), number of night shifts the past year as measured in 2018, and corresponding number of accidents at baseline (in 2016).
[2] The no change in QRs category includes those who had an equal number of QRs (+- 4) in 2016 and 2018.
